# A macrophage is a macrophage is a macrophage—in metastasis

**DOI:** 10.18632/oncotarget.28423

**Published:** 2023-06-06

**Authors:** Thomas T. Tapmeier

**Keywords:** cancer, metastasis, macrophages, melanoma, CCR

Macrophages have important roles in the response to infection or injury and can orchestrate the appropriate response after sampling their microenvironment, devouring anything untoward and presenting ingested antigens to T cells to elicit an adaptive immune response. In adult life, they develop from bone marrow-derived precursors and circulating monocytes, which differentiate into macrophages within tissue [1]. Apart from their role in clearing challenges to tissue integrity, macrophages have an essential role in growth-related processes such as angiogenesis and vascular remodelling, neural patterning, and ductal growth of developing glands [2].

However, their powers can be usurped by tumours, which cannot grow beyond a certain size or metastasis without the help of macrophages [3]. This is crucial, as the primary tumour might be amenable to treatment—so that patients can live with it—but metastasis is yet untreatable and inevitably becomes incompatible with survival.

Macrophages perform a range of physiological functions and are able to activate function-specific gene repertoires; however, surface markers for selectively targeting macrophages of one or another function are still elusive, despite recent advances in the field [4]. Thus, therapy attempts based on countering macrophages try to target the surface receptors that recruit them to sites of infection, inflammation or growth [5]. These receptors are known as chemokine receptors (CCRs), and each of these pairs with chemokine ligand molecules (CCLs) which fit into the receptors like keys into a lock [6]. A trail of ligand molecules can attract macrophages to the sites where they are needed. The activation of CCRs happens in cascades, with different CCL/CCR-pairings taking over during the time of the evolving macrophage activation scenario [7].

Clinical trials involving CCL/CCR inhibition—thought of as a new tool to augment therapy options—are largely promising [8]. However, with macrophages comprising diverse populations with different and at times opposing functions, we wondered how resident versus infiltrating cells of the myeloid lineage would respond to metastatic challenge, and how the inhibition of CCL/CCR signalling would influence the outcome.

We had previously shown the CCL2/CCR-2 axis as pivotal in recruiting monocytes to enable liver metastasis from colorectal cancer [9]. Thus, we now addressed the question of how lung macrophages would evolve during metastatic growth of lung colonies in a mouse model of melanoma, and what the results of CCR inhibition would be during the establishment and growth phases of these colonies [10].

Macrophages are of the myeloid (i.e., marrow-derived) lineage of white blood cells and, in the lung, broadly fall into two categories: The resident alveolar macrophages—there from birth and guarding the lungs from within—and infiltrating monocytes; cells recruited from the blood stream and ultimately from the bone marrow that convert into full-blown macrophages upon arrival in the lung. These two populations can be distinguished by the expression of integrin surface markers: Alveolar macrophages are F4/80^+^CD11c^+^, infiltrating monocytes are F4/80^+^CD11b^+^. However, their functional phenotype is not flagged by markers such as these, and because of the ambiguity of macrophage surface markers, we first defined gene expression signatures of pro- and anti-tumourigenic macrophage populations *in vitro* and compared these to tumour-associated macrophages (TAM) isolated from melanoma. Armed with these data, we interrogated the changes in gene expression in F4/80^+^CD11c^+^ alveolar (resident) macrophages in the lung and in the infiltrating F4/80^+^CD11b^+^ monocyte population summoned there through CCL/CCR signalling, and we compared the gene expression not only between these two populations but, crucially, between an early time point (day 3 after injection of melanoma cells) and a late time point (day 21). Over the time of metastasis, the infiltrating population increased in percentage and made up the majority of lung macrophages by the late time point. The analysis of gene expression patterns showed a conversion of the infiltrating F4/80^+^CD11b^+^ monocyte population from an initial inflammatory and anti-tumourigenic phenotype to a late wound healing, pro-tumourigenic phenotype, akin to TAM. Interestingly, the F4/80^+^CD11c^+^ alveolar macrophages did not convert in this fashion.

We noticed that the expression of CCR receptors 1, 2 and 5 was prominently upregulated in both macrophage populations by day 21 in response to the tumour burden, while our *in vitro* data told us that the melanoma cells (B16F10) made large amounts of the cognate CCL5 ligand (also known as RANTES). We thus decided to see what would happen if this signalling pathway was interrupted: We repeated the lung metastasis experiment but this time injected specific CCR inhibitors against CCR-1, CCR-2 or CCR-5 into the mice before and during the metastatic growth. Interestingly, the inhibition of CCR-1 decreased the metastatic burden significantly. Even more interesting, though, was the finding that the inhibition of CCR-2 *increased* the metastatic burden in mice – while CCR-5 inhibition did not have any apparent effect on metastasis. Our analysis of the macrophage populations within the lungs of these mice by flow cytometry showed that the inhibition of CCR-1 decreased the recruitment of the anti-inflammatory/pro-tumoural F4/80^+^CD11b^+^Ly-6C^−^ population of infiltrating monocytes and thus enabled the resident F4/80^+^CD11c^+^ alveolar macrophages to expand—and protect the lung from tumour growth. On the other hand, inhibiting CCR-2 reduced the recruitment of the beneficial inflammatory/anti-tumoural F4/80^+^CD11b^+^Ly-6C^+^ monocytes; cells that would have fought tumour growth but did not get the chance—with sorry results for the mice.

We hope that these results will inform future attempts at anti-macrophage therapy and lead to better outcomes for patients in the not-too-distant future.
